# A Survey of Reported Practices and Values of Smaller-Scale Dog Breeders in Canada

**DOI:** 10.3390/ani16142203

**Published:** 2026-07-15

**Authors:** Georgia Ruyter, Alexandra Protopopova, Quinn Rausch

**Affiliations:** 1 Animal Welfare Program, Faculty of Land and Food Systems, The University of British Columbia, Vancouver, BC V6T 1Z4, Canada; a.protopopova@ubc.ca; 2 Messerli Research Institute for Human-Animal Interaction, University of Veterinary Medicine Vienna, 1210 Vienna, Austria; quinnrausch96@gmail.com

**Keywords:** dog breeding, animal welfare, management, small-scale breeders, survey research

## Abstract

Dog breeding has received increasing attention due to concerns about animal welfare and the ethics of companion animal commercialization, yet smaller-scale breeders remain underrepresented in research despite potentially operating under different conditions. A survey of responding Canadian dog breeders (*n* = 464) provided quantitative and qualitative data on demographics, housing and breeding practices, and perspectives on welfare and regulation; quantitative responses were analyzed descriptively and with latent class analysis, and open-ended responses were thematically coded. Most participants were small-scale breeders who reported keeping dogs in home environments, averaging 3.28 breeding dams annually and maintaining relatively low caretaker-to-dog ratios compared to previously studied large-scale operations, while reporting frequent use of enrichment, socialization, and training practices. Qualitative findings indicated that responding breeders emphasized the importance of early-life care and responsible ownership, and expressed concerns that regulations and public discourse often do not reflect small-scale breeding contexts. Participants also identified broader welfare risks within the industry, including overbreeding and inadequate health and behavioural care. Overall, these findings suggest that small-scale breeding represents a distinct model within the Canadian dog breeding sector and highlight the importance of incorporating breeder perspectives when developing welfare policies and future research priorities.

## Simple Summary

Concerns surrounding dog welfare have sparked debate about breeding practices, but we still know little about the different types of breeders and how they manage their dogs. Breeders that responded to our national survey reported operating on a small scale, keeping only a few dogs and emphasizing socialization, training, and individualized care in their practices. While breeders generally reported that maintaining high welfare standards was important to them, many expressed that existing regulations do not reflect the realities of small-scale breeding and that public discourse often overlooks their efforts. In addition, although breeders acknowledged potential risks to dog welfare, they reported engaging with practices they believed supported dog wellbeing. Overall, our findings from a self-selected population suggest that small-scale home breeding represents a distinct model within the Canadian dog breeding sector, characterized by hands-on management and a stated commitment to welfare. We suggest that recognizing this diversity, alongside breeders’ perspectives, may support the development of effective welfare policies.

## 1. Introduction

In Canada, 56% of households own some type of companion animal, of which dogs and cats make up a large majority [1]. Canadians acquire dogs from a variety of sources, and dog breeders represent the most common source of acquisition, accounting for the largest proportion [1]. The exact proportion of dogs that are purebred in Canada remains unknown. However, because breeders are the most common source of dogs in Canada, their practices and values are of particular importance, as they are responsible for supplying the majority of dogs to Canadian households.

Over the past few decades, criticism of pedigree breeding has emerged within academic and veterinary communities as well as among the public and the media. Concerns about dog breeding include the high prevalence of inherited disease as well as housing and management conditions of dogs. Given the reported range of possible living conditions, the welfare of breeding stock is particularly important as these individuals may be subject to repeated pregnancies, social isolation, restrictive housing conditions, and limited opportunities for enrichment, all of which have significant impacts on dogs’ physical and psychological health. Much of the existing literature on the care, housing, and management of breeding stock centres around dogs housed in large-scale breeding operations. These studies typically emphasize the welfare challenges associated with high-density housing, limited socialization, inadequate veterinary care, and insufficient environmental enrichment [2].

One set of management practices that has received particular attention involves kennel housing conditions, where spatial confinement and limited socialization are features often associated with commercial facilities. These features lead to fearfulness, which reduces dog welfare [3]. Dogs living in kennel housing often develop atypical behaviours characteristic of extreme fear or shyness, abnormal aggression, or acute stereotypies [4]. The common causes of these problem behaviours may include stress-induced psychopathology and the absence of early socialization and exposure to novelty or common household stimuli [5]. Socialization [6] is limited in kennel environments due to the short daily care interactions with people and lack of opportunities for interactions with conspecifics [7]. Studies in laboratory environments have shown that dogs housed with conspecifics exhibit fewer abnormal behaviours and lower stress levels than those housed alone, indicating the importance of intraspecific social contact [8]. Puppies in particular are sensitive to early social experiences; limited exposure to humans or other dogs during critical developmental periods has been associated with increased fearfulness, poor adaptability, and long-term behavioural challenges [9]. Dogs raised in environments that limit interaction due to spatial confinement, lack of staffing, or restrictive management practices may be at increased risk for chronic stress and behavioural inhibition [2,10]. Opportunities for regular affiliative contact with both humans and other dogs should therefore be considered an essential component of breeding practices, with relevance to both adult breeding dogs and developing puppies. Because research has found that housing conditions impact dog welfare, larger-volume dog breeding facilities are regulated; however, we do not know whether and how housing methods differ in smaller-scale breeders.

Previous research using direct observation in the United States in legal commercial breeding kennels has suggested that dog breeding practices may vary, and that the nature and degree to which dogs may suffer in breeding facilities are related to their management [11]. Canada’s regulatory, geographical and cultural differences from the United States may further broaden this variation in dog management practices. As such, understanding management practices of breeders beyond large-scale commercial American facilities may provide a proxy to understanding the welfare of dogs in various types of breeding environments.

Dog breeding remains a primary source of companion animals, yet limited research has examined the daily management and housing conditions that shape the welfare of breeding dogs, particularly outside large-scale commercial operations. Although this study was primarily exploratory and did not test formal hypotheses, a questionnaire was developed to address three guiding research questions: (1) how Canadian dog breeders house and manage their dogs, (2) how these practices compare with those reported in the commercial breeding literature, and (3) whether distinct breeder profiles could be identified based on patterns of management practices, housing, and breeder characteristics. This study aimed to provide an empirical, data-driven understanding of dog breeding practices in Canada, with a focus on housing, husbandry, and breeder perspectives across a range of operational scales. Using survey data from Canadian breeders, this research documented management practices, compared them to those reported in commercial breeding literature, and explored whether distinct breeder profiles could be identified. The findings indicate that most participants reported operating at a small scale, with practices centred on home-based environments, individualized care, and frequent use of socialization, enrichment, and training. Compared to large-scale commercial systems, these breeders reported lower dog-to-caretaker ratios and greater integration of dogs into daily human activities. Patterns in responses further suggested meaningful variation across breeders, supporting the existence of distinct management approaches within the Canadian context. Overall, the results highlight that small-scale breeding represents a distinct and understudied model, emphasizing the importance of incorporating diverse breeding contexts into research, policy discussions, and the development of welfare standards.

## 2. Materials and Methods

### 2.1. Survey Design

The online survey was administered using Qualtrics© Provo, UT, USA (2022). All responses were collected anonymously, and participants had the option to choose to provide contact information for future research opportunities. The survey took a mean of 38 min to complete. The survey was designed to capture a comprehensive picture of housing, management practices, motivations, and attitudes of individuals involved in dog breeding in Canada. Participation was limited to those who reported having purposely bred one or more female dogs (dams) in the past three years. Individuals who indicated that their breeding was accidental, that they had only bred sires, or that they had not bred dogs during this period were excluded from the remainder of the survey. The survey was composed of four main sections: Size and Management, Housing and Daily Care, Attitudes and Values, and Breeder Demographics. Both quantitative and qualitative questions were included to allow for descriptive statistics and latent class analyses (LCA), as well as thematic coding of open-text responses. In total, the survey contained 58 questions, including 10 open-ended items, 4 Likert-scale questions, and 2 ranking questions, with the remainder in multiple-choice format. The full survey instrument is provided in Appendix A.

The Size and Management Section contained twenty open-ended questions producing numerical responses such as counts. Respondents were asked to report the number of breeding females, intact males, retired dogs, and litters produced each year between 2022 and 2024. They were asked about the number of breeds or crossbreeds they had produced, whether their primary breed was a crossbreed, and the specific breed they had been most involved with. A series of 10 multiple-choice questions addressed formal practices, including record-keeping, club membership, and use of genetic testing. Participants also identified their preferred breeding techniques (e.g., natural, transcervical, surgical insemination) and the typical veterinary care provided to breeding females, which produced categorical data. One Likert-scale question asked respondents about limitations to increasing scale of operations, ranging from lack of space, time, resources and due to regulations, which produced ordinal data.

The Housing and Daily Care Section contained 15 questions, including two frequency-based items (e.g., how often specific practices were performed, with response options ranging from daily to never) and one numerical write-in item, with the remainder in multiple-choice format. This section produced primarily categorical data from multiple-choice questions describing housing types, enrichment, and care routines, as well as ordinal data from Likert scales for identified limitations to increasing operation scale and frequency items measuring how often specific practices were used, and one numerical item from the write-in response where respondents wrote in an age range. Data was gathered on the number of regular caregivers, housing arrangements for breeding dogs (e.g., in the home, kennel, or foster homes), and the extent of dogs’ access to human interaction. Participants identified where dams typically whelped and where they were housed with their litters during the nursing period. They were also asked whether dams could voluntarily leave their puppies during rearing and whether females were regularly taken off the property for enrichment. To assess regulatory context and operational constraints, participants indicated which care standards they followed (e.g., Canadian Kennel Club, Canadian Veterinary Medical Association, self-designed protocols), whether those pre-established standards posed any practical challenges, and the extent to which factors such as time, space, and regulations limited their ability to expand the scale of their breeding operation. They were also asked to estimate the percentage of buyers who visited their facility in person and to describe any challenges associated with those visits. Participants were also asked about the routine training and enrichment practices in their kennel using a frequency scale (daily, several times a week, weekly, monthly, never). Training items included leash walking, obedience, crate training, house training, tricks, and professional or sport-specific training. Enrichment questions assessed the frequency of use of toys, chews, and puzzle feeders and interaction with humans, other dogs, or other animals. Several questions explored return policies and rehoming practices for retired dogs. Reproductive practices were explored further through questions about the age range at which breeding typically begins, the frequency of medical interventions during birth, inter-litter intervals, and the reasons dams were retired from breeding.

The Attitudes and Values Section was made up of 9 questions, consisting of three ranking items, three open-ended free response items, and 1 Likert-scale question, with the remainder in multiple-choice format. The ranking and Likert-scale questions produced ordinal data, while the multiple-choice items generated categorical data, and the open-ended questions provided qualitative data describing breeders’ perspectives in greater detail. This section included a series of questions on the intended purpose of the dogs being bred (e.g., companionship, working or competition) and how breeders ranked the importance of various welfare priorities (e.g., physical health, temperament, enrichment, facility cleanliness). Additional Likert-scale questions assessed perspectives on the moral status of animals, the role of legislation and third-party monitoring, and the potential impact of regulations on dogs, breeders, and buyers. Breeders were also asked to rate how intelligent and sentient they believed dogs to be, on a scale from 0 (like an inanimate object) to 10 (like a human). Two open-ended questions invited participants to identify the greatest risks to dog welfare in breeding and to describe measures they take to address these concerns.

Finally, the Breeder Demographics Section had 14 questions: one open-ended item and the remainder in multiple-choice format to collect basic background information. Participants provided their location (country, province, and city if comfortable), rural or urban setting, and dwelling type. Additional questions addressed whether the respondent had an occupation outside of breeding, their experience and education in animal care, household income, age, and formal training in breeding or training.

### 2.2. Recruitment and Participants

Recruitment began by consulting with individual breeders, breed clubs, and relevant organizations in December 2023. The survey was distributed beginning in October 2024 and remained open until March 2025. Subsequent recruitment was conducted through a variety of channels, including social media platforms, direct emails and phone calls to breeders, breed clubs, pet insurance providers, sporting dog organizations, veterinary clinics, dog show organizers and word-of-mouth referrals from other breeders. Although these recruitment methods were intended to reach a broad range of breeders, they primarily relied on existing breeder networks, organizations, and online platforms. Consequently, breeders operating outside these channels may have had fewer opportunities to participate.

### 2.3. Data Preparation and Analysis

Quantitative responses were analyzed using descriptive statistics and LCA, while qualitative answers were inductively thematically coded to provide additional context and insight into breeder concerns and motivations.

All quantitative statistical analyses were conducted using R (version 4.3.1; R Core Team, 2023) within the RStudio integrated development environment (version 2023.06.2 + 561; RStudio Team, 2023). Data cleaning and recoding were performed to prepare the dataset for analysis, including the conversion of text-based numerical responses to numeric format and the removal of predominantly incomplete or ineligible cases. Specifically, 21 respondents were flagged as potential automated entries based on CAPTCHA scores and their responses were manually examined and 5 were excluded, as were rows with no recorded responses and entries in which only the first question was answered while the remainder of the survey was left blank, which totaled 54 entries. To focus the analysis on Canadian breeding practices, two respondents located in the United States were also excluded.

During data cleaning, non-numeric entries for quantitative variables (e.g., responses recorded as words such as “two” or “four”) were recoded into numeric form to allow for quantitative analysis. This recoding was applied to several variables, including counts of adult females, adult males, retired dogs, and litters across the years 2022–2024. Extreme values were evaluated using visual inspection (histograms, boxplots) and normality tests (Shapiro–Wilk). After removing two extreme outliers reporting minimum breeding ages of 240 and 600 months, the dataset was adjusted accordingly, leaving a final sample of 343 respondents. In contrast, very low reported minimum breeding ages (e.g., 0 or 6 months) were more difficult to classify definitively as outliers, as respondents may have intended to report an age range rather than a specific value. Additionally, breeds vary substantially in the age at which they reach sexual maturity, limiting the precision with which minimum breeding age responses could be interpreted and warranting caution in drawing conclusions from this variable. All variables were converted into the appropriate data type (categorical or numeric) to facilitate subsequent statistical analyses.

The ratio of dogs to caretakers was calculated to provide an indirect measure of the intensity of animal management within each breeding operation. This variable was derived by combining respondents’ reported numbers of breeding females, breeding males (sires), and retired dogs to estimate the total number of dogs kept on-site. The number of regular caregivers was taken from a separate question asking how many individuals were routinely involved in the daily care of dogs.

### 2.4. Data Preparation for Latent Class Analysis

Patterns of responses to questions about breeder practices, training, and enrichment were identified by conducting LCA through the R package poLCA [12]. Thirteen variables were selected as indicators for the LCA model. Training, exercise, and enrichment activities were included because they reflect routine management practices of breeders that can influence dog welfare. These variables which were originally ordinal data (frequencies ranging from daily to 2–3 times per week, once a week, once a month and never) were recoded as binary indicators (1 = provided daily or two to three times per week, 0 = provided once a week or less, or never) as is typical for LCA modelling [13]. For some indicators, multiple related variables were combined to improve both theoretical coherence and model stability. For example, the “Regular Walks” indicator combined leashed and off-leash walks, and breeders were coded as 1 if either was provided at the specified frequency. Similarly, the “Training” indicator combined basic commands, tricks, and sports activities, and the “Physical Enrichment” indicator combined puzzle and chew activities. This approach addressed issues of low variation among individual items, which can reduce class separation and lead to unstable or non-identifiable models in LCA [14]. Grouping conceptually similar items also mitigated local dependence among highly correlated variables and enhanced the theoretical interpretability of resulting classes. Medical interventions were coded as 0 if breeders indicated “Never” and 1 if any other response was reported. Breeding styles, management and husbandry practices were also included, including compliance with CKC bylaws, dogs’ access to humans, whether dams could leave the property, and use of crates. Each was coded as a binary variable reflecting the presence (1) or absence (0) of the practice. Dog purposes were included as three separate binary indicators for companionship, working, and competition (1 = purpose applied, 0 = purpose not applied).

### 2.5. Descriptive Statistical Analysis

Initial data cleaning involved filtering the dataset to include only respondents who indicated that they had one or more breeding dams that they purposely bred. For numeric variables (e.g., number of dogs, litters, years of experience, age, income, importance ratings), measures of central tendency and dispersion (mean, median, standard deviation, and range) were reported, and visualizations such as histograms, boxplots, and density plots were used to explore their distributions. For categorical variables (e.g., dwelling type, intended breeding purposes, kennel club affiliation, housing practices, and agreement with attitudinal statements), frequency counts and percents were calculated, and tables and bar plots were created. For questions that included an ‘other (please specify)’ option, all responses were coded as ‘other’ regardless of whether additional detail was provided. This ensured that participants had the opportunity to indicate if none of the listed categories applied. In the analysis, ‘other’ responses were retained as a separate category in the descriptive statistics.

### 2.6. Latent Class Analysis

An LCA was conducted to identify subgroups of breeders with similar patterns of responses on key management and care variables. The analysis aimed to uncover underlying breeder typologies based on reported practices, rather than assuming uniform responses across the sample. A series of models specifying one to five latent classes were estimated and compared using fit indices, including the Akaike information criterion (AIC), Bayesian information criterion (BIC), and sample-size-adjusted BIC. Model selection also considered interpretability, parsimony, and relative entropy to determine the optimal number of classes.

All indicator variables were categorical, and local independence was assumed. Class profiles were interpreted based on the probability of each response for the included indicators, and classes were labelled to reflect distinguishing patterns in management or care practices. Following identification of the final model, posterior class probabilities were used to assign individuals to their most likely latent class. All LCA models were estimated using the R package poLCA [15], with model comparison and diagnostics conducted using a combination of standard package outputs and custom scripts.

The poLCA package fits polytomous latent class models via maximum likelihood estimation using the expectation–maximization (EM) algorithm. By setting na.rm = FALSE, cases with partially missing manifest responses contributed to parameter estimation using all available data, rather than being discarded. This approach is analogous to full-information maximum likelihood (FIML), which does not impute missing data but uses all observed data, both complete and incomplete, to estimate model parameters. FIML has been shown to be an efficient method for handling missing data in LCA and is the preferred approach in most software packages for LCA [14].

Model selection was based on a combination of statistical fit and substantive interpretability. Lower AIC, BIC, and adjusted BIC values were considered indicative of improved model fit, while entropy was used to evaluate classification quality. Entropy values range from 0 to 1, with higher values indicating greater certainty in class assignment and clearer separation between latent classes. Because information criteria may continue to improve as additional classes are added, final model selection also considered whether additional classes represented substantively meaningful and distinct breeder profiles rather than minor subdivisions of existing groups. Posterior class membership probabilities were examined to assess classification certainty and to ensure that respondents could be assigned to latent classes with an acceptable degree of confidence. Individuals were assigned to the class for which they had the highest posterior probability, and average posterior probabilities were reviewed as an additional indicator of class separation and model adequacy.

### 2.7. Comparison to Commercial Breeding Operations

Drawing from Stella et al.’s [16] survey design, this study adapted a subset of their management questions related to enrichment, training, and exercise, three domains most relevant to the ongoing welfare of breeding adults, to investigate whether and how smaller-scale breeders implement these practices. See the Results Section for comparison. However, while their study included questions relevant to both puppies and adult dogs, this survey focused specifically on adult breeding stock in breeding contexts to reflect the scope of this study. For this survey, 10 of Stella et al.’s management questions were used, covering the use of basic commands, chews, leashed walks, play with other animals, play with dogs, play with humans, puzzle feeders, sports, toys, and tricks, asking breeders to report the frequency with which they used each intervention. Although frequency responses were collected to capture nuance, dichotomization was required for comparison with Stella et al. The selected cutoff reflects a conceptual distinction between routine management practices (daily or 2–3 times per week) and sporadic use (once a week or less), the latter being unlikely to meaningfully influence dogs’ day-to-day lived experience. Percentages were calculated for each practice, allowing direct comparison of reported use between our sample of breeders and the original study, highlighting where smaller-scale breeders reported higher or lower adoption of these management interventions.

### 2.8. Qualitative Coding

Qualitative responses were analyzed for two open-ended survey questions: “In your opinion, are there limitations to current standards that are difficult or illogical to follow? If yes, please describe how.” and “What do you think is the biggest risk to dog welfare in the dog breeding industry?” Each question was analyzed separately using inductive thematic analysis to identify common themes [17].

First, the responses were imported into Lumivero (2025) NVivo version 15 for coding. Each open-ended question was analyzed separately using inductive thematic analysis to identify common themes [18]. First, the first author (GR) and the secondary coder (QR) read through all responses and worked iteratively to develop an initial codebook for each question. The coders went back and forth to discuss emerging ideas, refine definitions, and reach agreement on a preliminary set of codes.

Once the initial codebooks were established, the author (GR) coded the full set of responses for each question. After this first round of coding was completed, the secondary coder (QR) independently coded all responses using the same codebooks. Following the independent coding, coding decisions were compared, discrepancies were identified, and coders met to discuss differences. Through a progressive process, moving back and forth between the coded data, the codebook, and coder interpretations, definitions were revised, limits between codes were clarified, and consensus was reached on all disagreements.

This process continued until full agreement was reached and the final codebooks were established, and all responses were double-coded. The complete codebooks, including all definitions, are provided in Appendix B (Table A1 and Table A2). Representative quotations selected for Section 3 were chosen based on their clarity and how strongly they represented the themes identified by the authors. A total of 464 respondents initiated the survey. Qualtrics’ automated quality checks were used to flag potential bot responses and surveys with substantial missing data. All flagged responses were subsequently reviewed manually, and only those that displayed characteristics consistent with automated responding (e.g., nonsensical text or implausible patterns) or early termination were removed. A total of 387 valid responses remained for analysis; of these, the majority reported having one or more breeding dams that they purposely bred (*n* = 347). Respondents who selected other options, such as indicating that they bred or allowed only a sire to breed (*n* = 25), had never bred dogs (*n* = 21), or experienced an unplanned litter (*n* = 8), were directed to the end of the survey and did not continue. Since each question was optional, some respondents did not participate or respond to all questions. The counts are listed, and percents represent percentages of participants who responded, not of the sample.

## 3. Results

### 3.1. Breeder Characteristics

Most breeders reported living on rural properties outside of concentrated housing areas (*n* = 148, 57.1%). Smaller proportions of breeders lived in small towns, small cities and large towns. The remaining counts and percentages are listed in Appendix D (Table A4).

Most breeders lived on acreage properties (*n* = 133, 51.4%) or in detached houses (*n* = 106, 40.9%). The remaining counts and percentages are listed in Appendix D (Table A5). Just under half reported having formal training in animal husbandry, medicine, or breeding (*n* = 117, 45.9%), while a slightly larger proportion did not have formal training (*n* = 138, 54.1%). Just over half reported having formal education in animal training (*n* = 134, 52.3%), while 122 breeders (47.7%) reported that they did not. Among 253 respondents, the mean years of experience was 21 (SD = 15), with a median of 18 years. Reported experience ranged from 1 to 69 years. See Appendix D (Table A6) for other demographic variables and results.

### 3.2. Size and Management

Across the three-year period (2022–2024), the average number of adult breeding females per breeder was 3.07 (SD = 3.23), with a median of 2.33 (*n* = 327). Most breeders maintained between one and three adult breeding females, as reflected by an interquartile range (IQR) of 2.00.

The average number of adult breeding males reported over the same period was lower, at 1.83 (SD = 1.79), with a median of 1.33 (*n* = 323). Most breeders reported maintaining between zero and three adult breeding males (IQR = 3.00).

The average number of retired dogs per breeder was 2.49 (SD = 2.99), with a median of 1.67 (*n* = 321). The interquartile range (0.67–3.00) indicated that most breeders housed between one and three retired dogs.

Breeding activity was further reflected in the mean number of litters produced per year, which was 1.46 (SD = 2.17) across respondents (*n* = 326). The median was 1 litter per year, indicating that the majority of breeders produced one to two litters annually.

Breeders reported keeping an average of 1.24 breeds or crossbreeds over the past three years (SD = 0.67, *n* = 330). The median number of breeds was 1 (IQR = 1), indicating that most breeders focused on a single breed. Values ranged from 0 to 6 breeds, reflecting limited multi-breed operations within the sample.

When asked whether their primary breed was a crossbreed, the majority of respondents (*n* = 314, 95.2%) reported “No,” while a small proportion (*n* = 16, 4.8%) identified their primary breed as a crossbreed. Five respondents did not provide a response.

The most frequently reported primary breeds were Labrador Retriever (*n* = 27, 8%), Australian Shepherd (*n* = 13, 4%), Golden Retriever (*n* = 11, 3%), Nova Scotia Duck Tolling Retriever (*n* = 9, 3%), Belgian Shepherd Dog (*n* = 8, 2%), Border Terrier (*n* = 8, 2%), and German Shepherd Dog (*n* = 8, 2%). Other breeds, including both popular recognized purebreds (e.g., French Bulldogs, Dachshunds, other bulldogs) and designer crossbreeds (e.g., Goldendoodle, Labradoodle, Bernedoodle), were reported at much lower frequencies.

A complete list of all reported breeds and their frequencies is provided in Appendix C (Table A3).

Breeders in our sample most commonly kept vaccine records (*n* = 320, 83%), genetic records (*n* = 303, 78%), reproductive history (*n* = 298, 77%), and sales records (*n* = 294, 76). Dewormer and flea/tick treatment records were also frequently maintained (*n* = 282, 73%), followed by import and export records (*n* = 185, 48%), observation sheets for behaviour (*n* = 160, 41%), tracking of food intake and/or elimination (*n* = 139, 36%), and other types of records (*n* = 125, 33%). Examples of other tracked records that breeders listed included performance history and puppy applications.

Many breeders reported belonging to the Canadian Kennel Club (*n* = 290, 88.2%), followed by breed-specific groups (*n* = 243, 73.9%). Fewer breeders participated in local breeding networks (*n* = 100, 30.4%) or other types of organizations (*n* = 92, 28.0%), such as training clubs. Only a small proportion of breeders indicated that they did not belong to any breed club (*n* = 10, 3.0%).

The majority of breeders reported that they routinely completed genetic testing (*n* = 306, 93.3%). A small number of breeders indicated that they only test if there is an issue with the dam’s or sire’s offspring (*n* = 11, 3.3%), and an equal number reported that they do not complete genetic testing at all (*n* = 11, 3.3%).

Breeders most commonly reported taking female dogs for emergency examinations when sick or injured (*n* = 303, 92.4%), routine preventive wellness examinations outside of pregnancy (*n* = 284, 86.6%), and legally required vaccinations (*n* = 280, 85.4%). Examinations and monitoring during a healthy pregnancy were also frequently reported (*n* = 261, 79.6%), as were examinations during pregnancies with health issues (*n* = 206, 62.8%). Optional vaccinations were less commonly reported (*n* = 177, 53.9%), and a smaller proportion of breeders indicated other reasons for veterinary visits (*n* = 72, 22.0%).

Most breeders reported using their own experience in designing housing and care programmes (*n* = 259, 79.0%) and/or following Canadian Kennel Club (CKC) bylaws (*n* = 210, 64.0%). Fewer breeders reported following the Canadian Veterinary Medical Association Code of Practice for Canadian Kennel Operations (*n* = 81, 24.7%) or other standards (*n* = 54, 16.5%).

**Figure 1:**
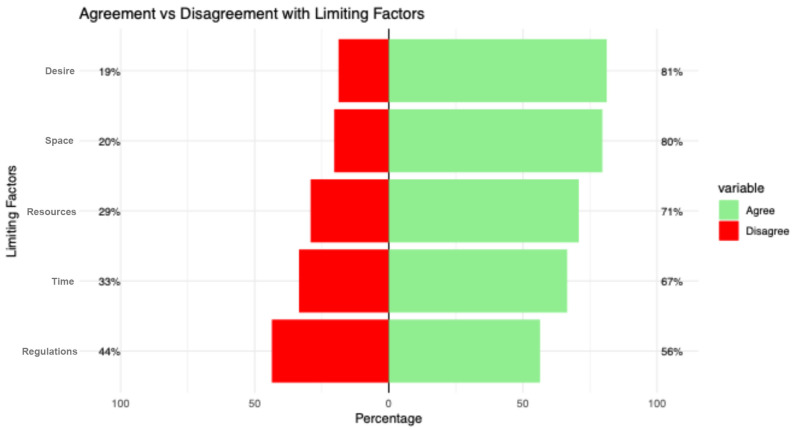
Participant agreement and disagreement (neutral responses excluded) regarding factors limiting breeding practices, collapsed across a five-point Likert scale into two categories (“Agree” and “Disagree”); neutral responses were excluded. Sample sizes therefore vary across items (*n* = 188–216).

Breeders reported a lack of desire as the main limiting factor to increasing the size of their operation (*n* = 176, 81%), followed by physical space (*n* = 177, 79%; Figure 1).

Breeders reported that, on average, 68.8% of buyers view their premises and meet their dogs before purchasing puppies (median = 80.0%), with a standard deviation of 33.6 and values ranging from 0% to 100%. The 25th and 75th percentiles were 50 and 100%, respectively, yielding an interquartile range of 50%. Overall, the distribution showed considerable variability (SD = 33.6%).

Most breeders indicated that there is no situation in which they would not accept the return of one of their dogs (*n* = 276, 89.3%). A small proportion reported that there are situations in which they would not accept a return (*n* = 27, 8.7%). Six respondents chose not to respond.

### 3.3. Housing and Daily Care

Breeders reported a mean of 2.45 caregivers (who regularly care for their dogs, including themselves) per operation (SD = 1.46), with a median of 2 caregivers, indicating that most operations are managed by a small number of people. Most reported home housing their dogs (Table 1).

Most breeders indicated that their breeding dogs spent most of their time in areas with free access to humans, such as being in the home as pets (*n* = 261, 86.4%). Fewer breeders reported that their dogs spent most of their time in areas with frequent access to humans (e.g., free ranging in a yard or garage/barn; *n* = 22, 7.3%) or in areas with controlled access to humans, such as kennels, runs, or crates (*n* = 19, 6.3%).

The majority of respondents (*n* = 272, 90.4%) reported that their dams gave birth within their own homes. A smaller proportion indicated that dams gave birth at a veterinary clinic (*n* = 10, 3.3%), at another location under the management of others such as guardian homes (*n* = 4, 1.4%), or in a separate building on their property that they do not live in (*n* = 3, 1.0%). No respondents reported births occurring outside, while 4% (*n* = 12) selected “Other.”

Most breeders reported that dams remained in their homes while nursing puppies (*n* = 289, 96.7%). A small proportion indicated that dams nursed in a separate building on their property that they did not live in (*n* = 7, 2.3%), while a few reported that dams were housed at another location under the management of others, such as a foster home (*n* = 3, 1%). No respondents indicated that dams nursed outside, at a veterinary clinic, or in another type of location.

Most breeders reported that nursing dams could leave their litters freely at all times (*n* = 274, 91.3%). A smaller proportion indicated that dams could leave only at certain times (*n* = 24, 8%), while very few reported that dams were not able to leave their puppies at all (*n* = 2, 0.7%).

Almost three-quarters of breeders reported that their dogs regularly left the property (e.g., walks, visits to the park; *n* = 219, 73.2%). Just over one-quarter indicated that their breeding females did not leave the property for enrichment (*n* = 80, 26.8%), with some providing reasons for this choice, citing concerns about disease transmission or the perception that off-property outings were unnecessary because their dogs had ample space to exercise and play on rural properties.

Table 2 shows the comparison of provided enrichment in the study population compared to the previously reported data in large-scale commercial facilities.

The minimum age at which breeders typically begin breeding their dams ranged from 0 to 64 months, with a mean of 24.9 months (SD = 8.2) and a median of 24 months (IQR = 3.8). The maximum age ranged from 0 to 108 months, with a mean of 58.7 months (SD = 23.4) and a median of 60 months (IQR = 24.0), indicating greater variability in the upper end of the breeding age range.

Just under half of breeders (*n* = 158, 54.9%) reported that medical interventions were sometimes needed during birth, such as induction or cesarian sections, while one-third (*n* = 98, 34%) indicated that interventions were never required. A smaller number of breeders reported that interventions were needed about half the time (*n* = 17, 5.9%), most of the time (*n* = 7, 2.4%), or always (*n* = 8, 2.8%).

Most breeders reported waiting until the second heat after whelping to breed their dams again (*n* = 110, 39.0%). The remaining counts and percentages for other responses are listed in Appendix D.

The most commonly reported reason for retiring dams from breeding was after having a certain number of litters (*n* = 227, 30.2%), followed by retirement at a certain age (*n* = 208, 27.6%). Behavioural, medical, or genetic issues were cited by breeders (*n* = 184, 24.5%), while some (*n* = 133, 18%) reported retiring dams on the advice of a veterinarian. Fewer breeders selected other reasons (*n* = 58, 7.7%).

Breeders who indicated that they retire dams after a certain number of litters reported a mean of 2.9 litters and a median of 3 litters (IQR = 1), indicating that most breeders retire dams after three litters.

One-fifth of breeders (*n* = 55, 20%) reported that they sell or rehome retired animals. A larger proportion (*n* = 129, 44.5%) indicated that they do not, while some (*n* = 106, 36.6%) selected Other.

### 3.4. Attitudes and Values

Respondents reported a range of primary purposes for which they bred dogs, listed in Table 3.

Breeders ranked the importance (from 1 being the least to 10 being most important) of five aspects of dog welfare: physical health, mental wellbeing, genetic health, conformation, and temperament. Summary statistics for each welfare aspect are provided in Table 4. Table 5 presents the summary statistics for breeder rankings of housing and welfare aspects.

**Figure 2:**
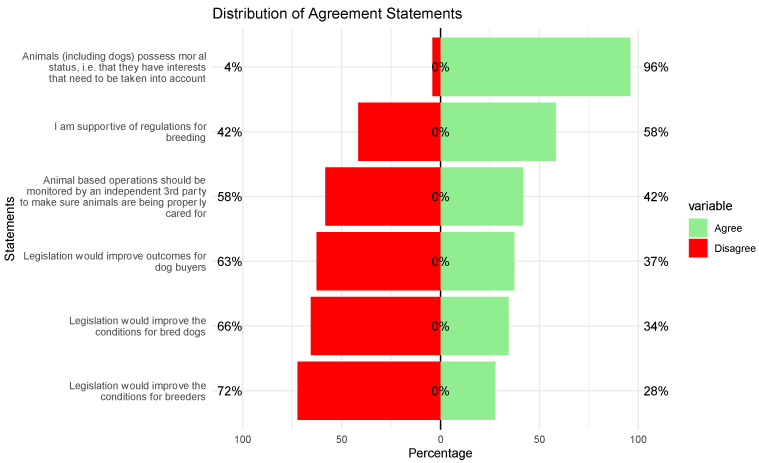
Participant agreement and disagreement (neutral responses excluded) in response to statements regarding animal welfare, regulation, and legislation within the Canadian dog breeding industry, collapsed across a five-point Likert scale into two categories (“Agree” and “Disagree”) (*n* = 252).

Breeders reported a mean sentience rating (i.e., aware or conscious) on a scale from 0 (“like an inanimate object”) to 10 (“like a human”) of 7.98 (SD = 1.59) and a median of 8. Reported values ranged from 0 to 10, though most respondents clustered toward the upper end of the scale (Figure 2).

Among breeders with available data, the mean caregiver-to-dog ratio was 2.84 dogs per caregiver, corresponding to approximately one caregiver for every three dogs. Caregiver-to-dog ratios varied across breeders, with values ranging from 0.06 to 14.3 dogs per caregiver.

### 3.5. Latent Class Analysis

In LCA, the number of model parameters increases with the number of classes specified. To evaluate whether the sample size is sufficient for each model, the ratio of sample size to estimated parameters was calculated. This is a commonly used rule of thumb, where a ratio greater than 5 is considered acceptable for stable model estimation. The code that was used iteratively fits models with two to five classes, estimates the number of parameters for each solution, and then divides the sample size by the parameter count.

From these results, the two-class to five-class solutions all meet the recommended threshold, with the five-class solution reaching the limit. This suggested that, based on sample size considerations, the 2–5-class solutions may be appropriate for interpretation in this dataset (Table 6).

The number of latent classes was increased sequentially, beginning with the one-class solution until the five-class model (where the sample size per parameter limit was established), and the best-fitting model was identified [15]. Although the one-class model had the lowest AIC and adjusted BIC values, it was not considered a meaningful representation of heterogeneity, as all breeders were assumed to belong to a single, homogeneous group, and entropy was undefined (∞), indicating that there was no meaningful classification.

Models specifying 2–5 classes were then compared. Fit statistics, including AIC, BIC, adjusted BIC, and entropy, were considered alongside the theoretical interpretability of the classes and the clarity of classification. The two-class solution was selected as the best-fitting model, as it provided the lowest AIC and BIC values as well as higher entropy (0.669), indicating clearer separation of respondents into distinct classes.

Although models with three to five classes were examined, they did not improve overall model fit. The two-class solution had the lowest AIC (3435.69), BIC (3539.47), and adjusted BIC (3453.81) among all multi-class models. It also demonstrated the highest entropy (0.669), indicating better class separation than the three-class (0.615), four-class (0.518), and five-class (0.550) solutions. While the entropy value suggested moderate rather than perfect classification certainty, it provided the clearest distinction between breeder groups among the candidate models.

In addition to statistical fit, the two-class solution was selected because it yielded substantively interpretable classes that differed in their engagement with formal breeding institutions and management practices. Models with additional classes primarily subdivided respondents into smaller groups without generating clearly distinct or theoretically meaningful breeder profiles. Consequently, the two-class solution was judged to provide the most parsimonious and interpretable representation of heterogeneity within the sample.

**Figure 3:**
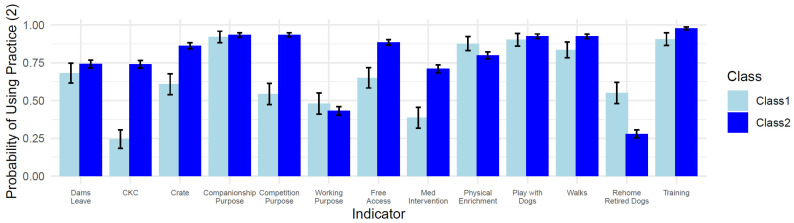
Item-response probabilities (±standard error) for breeding and management practice indicators derived from the two-class LCA model (*n* = 345). Bars represent the estimated probability that respondents within each latent class endorsed each item. Error bars reflect the uncertainty around each estimated probability. Differences in item-response probabilities across classes illustrate distinct patterns of breeding and management practices captured by the model.

Posterior probabilities were examined to assess classification quality prior to modal class assignment. Although some uncertainty remained, particularly for the smaller class, the posterior probability structure supported meaningful differentiation between the two identified breeder groups. Respondents were subsequently assigned to the class for which they had the highest posterior probability [15] (Figure 3).

#### 3.5.1. Class 1: Independent Breeders

Breeders in the Independent Breeders (*n* = 50) class had lower probabilities of endorsing several structured management practices compared to Structured Competition Breeders. Specifically, they were less likely to report following CKC bylaws (0.32 ± 0.03 vs. 0.79 ± 0.03) and using crates (0.73 ± 0.03 vs. 0.86 ± 0.03). In contrast, they were more likely to rehome retired dogs (0.49 ± 0.03 vs. 0.26 ± 0.03). Other practices, such as allowing dams to leave the property, taking regular walks, training, play with dogs, seeking medical intervention during birth, providing free access to humans, and breeding for companionship or working purposes, were reported at similarly high rates across both classes.

#### 3.5.2. Class 2: Structured Competition Breeders

Breeders in the Structured Competition Breeders class (*n* = 295) were more likely to be involved in formal breeding institutions like kennel clubs and were more likely to list breeding for competitions as their purpose (0.96 ± 0.03 vs. 0.65 ± 0.03 in Class 1). They were also more likely to report crate use. Other practices, such as regular walks, training, play with dogs, physical enrichment, breeding for companionship, and working as a breeding purpose, were similarly common across both classes.

### 3.6. Qualitative Analysis

#### 3.6.1. Perceived Risks to Dog Welfare in the Breeding Industry

Inductive thematic analysis of responses to the question “*What do you think is the biggest risk to dog welfare in the dog breeding industry?*” resulted in 15 themes, described below along with the count for each theme. Complete definitions and subcodes are provided in Appendix B.

*Animal advocacy*: The animal advocacy theme was identified in 32 responses. Participants discussed concerns related to external advocacy messaging affecting the industry, including stances such as believing that dogs should not be pets or the “adopt don’t shop” (P126) narrative. Some respondents believed that these perspectives harmed breeders or welfare overall. For example, one participant stated that “animal rights activists… take things to extremes.” (P27).

*Ineffective regulations or legislation*: This theme appeared in 60 responses. Participants expressed that regulations either fail to address harmful breeders and/or disproportionately burden ethical operations. Responses ranged from concerns about insufficient enforcement to frustration with regulation that favours large-scale operations. For example, one respondent noted “regulations… don’t prevent bad breeders and bad practices.” (P16).

*Extreme conformation in breed standards*: The concern that breed standards encourage unhealthy physical conformation emerged in four responses. Participants referenced specific issues, for example “Dog shows creating standards that are unhealthy for the dog, like German shepherds having back issues or Brachiocephalic dogs who can’t breathe well or give birth naturally.” (P48).

*Conflict between breeders*: This theme appeared in two responses. Some participants highlighted interpersonal hostility or disagreement within the breeding community, particularly regarding breeding philosophies (such as pedigree breeding vs. crossbreeding) or accepted practices.

*Breeders prioritizing profit:* Identified in 170 responses, participants described concerns about breeders prioritizing profit or meeting public demand over breed preservation or dog welfare. Examples ranged from identifying breeding systems like puppy mills or backyard breeders to more general descriptors like “high volume operations” to “greed” and overbreeding.

*Poor breeding stock selection*: This theme appeared in 156 responses. Participants cited insufficient genetic testing, knowingly breeding hereditary problems, or selecting breeding stock without sufficient health or temperament considerations. Many respondents also mentioned issues with crossbreeds and ‘designer mixes’ that fell into this theme.

*Overpopulation:* This theme was found in four responses. Participants listed breeding dogs beyond the need or demand contributing to an overpopulation of pet dogs. For example, one respondent wrote “Over breeding beyond the need for dogs…” (P106).

*Lack of accurate knowledge or understanding:* The concern that misinformation or lack of education affects welfare was mentioned in 131 responses. Participants described uneducated breeders, misinformed buyers, disagreements with the term industry, and misleading marketing by both breeders and rescues. More generally, some quotes reported an issue with misinformed or inaccurate public attitudes that shape supply and demand of dogs.

*Lack of access to veterinary care:* This theme emerged in 33 responses, with participants citing limited access to reproductive specialists and increasing veterinary costs.

*Failure to provide adequate daily care:* Responses in 52 entries referenced inadequate housing or environment, poor nutrition, and lack of regular enrichment or stimulation.

*Failure to commit to dogs for their whole life:* Identified in 32 responses, participants described the risk of dogs being unsupported by breeders after sale and the potential of dogs being unwanted by their owners and ending up in shelters.

Less frequently, participants highlighted welfare risks related to *importing dogs* (nine responses; disease and inadequate documentation), *the welfare of breeding dogs themselves* (10 responses; quality of life and challenges during pregnancy or whelping), *economic pressures affecting care and veterinary access* (two responses), and the *complexity* of welfare risks as shaped by interacting factors (one response).

*Unclear:* A subset of responses (23) lacked clarity and could not be reliably categorized.

#### 3.6.2. Limitations of Current Dog Breeding Standards

Analysis of responses to the question “*In your opinion, are there limitations to current standards that are difficult or illogical to follow? If yes, please describe how*” resulted in 10 final themes, described below along with their counts.

*Standards made for kennel operations are not applicable for home breeders:* Identified in 34 responses, participants felt that standards were designed for commercial-scale kennels rather than small home breeders. Some noted that biosecurity-focused design did not reflect realistic home environments and that some standards are too challenging to follow at home. For example, “Most do not apply to me, my dogs live in my home not a ‘facility’” (P16).

*One-size-fits-all standards are not always applicable:* Present in 19 responses, participants stated that standards failed to account for differences among breeders, breeds, litters, and individual dogs. Others described blanket bans (e.g., docking/cropping) that they felt were overly broad.

*Municipal bylaw constraints:* Reported in 42 responses, participants commented that local bylaws restricted dog numbers or that they found it difficult or costly to obtain kennel licences.

*Import/export restrictions*: This was discussed by six of the participants, who cited difficulties complying with import or export regulations (e.g., CDC and CFIA restrictions).

*Requirements without accessibility:* Identified in five responses, participants noted that standards may require health/veterinary testing that is expensive or takes too long and delays their breeding schedules.

*Standards don’t do enough:* Seen in 11 responses, some participants felt that current standards were insufficient or that they lacked meaningful enforcement, particularly regarding “backyard breeders.”

*Standards are incorrect:* Reported by one, this respondent believed that certain recommended practices were misguided or unsuitable.

*Criticism of organizations who create standards:* Appearing in seven responses, participants expressed frustration with the organizations developing standards (e.g., CKC, CVMA).

*Lack of understanding:* Identified in 18 responses, quotes reflected uncertainty about what standards exist, confusion regarding their meaning, or admission of not having read them.

*No limitations of standards:* 50 participants explicitly stated that there were no limitations to current standards.

*Uncoded:* Six responses were unclear or could not be categorized without assumptions: for example, “Colour that is naturally occurring in the breed that is Has no health issues attached and nothing more than visual.” (P255).

## 4. Discussion

While previous research has, at times, conceptualized breeders as a relatively homogeneous group, frequently using commercial breeding as the primary point of comparison, our LCA demonstrated variation within small-scale breeding, revealing two distinct groups. The variation between the two breeder classes identified by our LCA indicates that even within the home small-scale breeding community, there is no single, unified understanding of what makes a breeder “ethical” or “responsible”. The class of participants that we labelled *Independent Breeders* was less likely to follow CKC bylaws, list competition as a purpose for breeding, or use housing practices like crating. *Structured Competition Breeders* aligned more closely with formal guidelines and kennel club standards and reported higher use of crates. The practical significance of these differences lies less in individual practices and more in how breeders position themselves relative to established norms of responsible breeding. Breeders in the less structured class were not necessarily providing poorer care; rather, they were less likely to participate in kennel club systems, competitive events, and certain management practices commonly associated with formal breeding networks. Our sample includes a notably higher proportion of Structured Competition Breeders, who are highly involved in kennel clubs and competitions. Kennel clubs often advertise themselves to the public as a resource to find a reputable or ethical breeder [19]; however, many breeders around the world are moving away from and operating outside of the purview of traditional kennel clubs [20] and therefore cannot be vetted or verified through these channels. Recent evidence further suggests that relying on kennel clubs to drive widespread improvements in breeding dog welfare may be insufficient. While pedigree registries continue to shape perceptions of responsible breeding, their influence is diminishing as more dogs are bred and sold outside of formal club affiliations. Data from Denmark indicate that the majority of dogs are now acquired without kennel club registration, reflecting a broader trend toward decentralization of dog breeding [20]. In Canada, it is difficult to determine whether a similar trend exists based on this study because much of the recruitment relied on kennel clubs and breed-specific groups. It is therefore possible that a comparable shift away from kennel clubs is occurring in Canada, but this skewed sample could not definitively capture this.

Despite class differences, both groups reported predominantly home-based housing and frequent human interaction, exercise, and enrichment, suggesting broad agreement on the importance of behavioural and social care. Compared with previously studied commercial breeding kennels, participants in this study reported substantially greater use of training, exercise, and enrichment practices. Debates about what constitutes “ethical” breeding are shaped by differing values, priorities, and interpretations of risk. Within this study, breeders varied in how they defined threats to dog welfare and how they responded to them. Overall, these findings suggest that although breeders may share broad commitments to dog welfare, they differ in how welfare risks are defined, prioritized, and distributed across the breeding and ownership process. These differences echo the observation by Westgarth et al. [21] observation that “responsible dog ownership” is understood in varied and sometimes conflicting ways. When the public was asked what responsible ownership entails, owners emphasized contradictory points such as freedom vs. control, affection vs. discipline, prioritizing the dog’s needs vs. public safety, and relying on personal judgement even when their practices contradict others’ views of what responsibility entails. Together, these views show that, much like pet owners in the Westgarth et al. study, breeders interpret responsibility differently depending on how they see their role in a dog’s life. Collectively, these responses show that even when breeders agree on the importance of dog welfare, they diverge in how risks are defined, prioritized, and addressed.

These differing perspectives on responsibility and welfare risk provide a useful bridge to understanding how the dog breeding sector negotiates public trust and its broader social licence to operate. Hampton [22] argues that industries maintain their social licence to operate when they adopt a proactive, transparent approach to animal welfare, one that includes regular reporting of welfare outcomes and, ideally, independent oversight; a good example is the dairy industry, which has had long-standing engagement with animal welfare science [22]. Transparency, even when inconvenient or costly, is positioned as essential for sustaining public trust, while secrecy or reactive, crisis-driven reforms tend to erode it. Within this broader understanding of how animal-use sectors retain public acceptance, breeders’ views on regulation in the present study reveal a similar tension. While some breeders mentioned in open responses that they feel industry is not the appropriate term or label for dog breeding, they nonetheless operate like any other industry in relation to maintaining the public’s trust and their social licence to operate.

Other stakeholders, such as veterinarians, should also be included in discussions of breeding practices, particularly given that access to veterinary care was frequently reported by breeders in our sample as a major risk to dog welfare. For example, when asked to identify current welfare risks in the industry, respondents commonly cited “the lack of veterinarians and the cost of veterinarian care”. Veterinarians have also been prominent critics in debates surrounding pedigree breeding, as they are the ones who routinely see pet dogs affected by inherited disorders and breed-associated disease. Despite this, veterinary recommendations appear to be underutilized by breeders when it comes to management, with few breeders reporting that they follow the CVMA’s housing and management guidelines. This may also be due to the fact that the CVMA housing and management guidelines were not relevant to many of our breeders or that they are more applicable to kennel-style operations, as seen in the qualitative responses for limitations of current standards.

Participants also voiced concern regarding inadequate breeding stock selection, including lack of genetic testing or validation of health or temperament prior to breeding. Interestingly, respondents described these issues as perpetuated not only by “irresponsible” breeders but also by uninformed buyers. This highlights a prominent ethical challenge, which is that increasingly, breeders may be unable to maintain high standards without consumer demand for these practices. Public misunderstanding, particularly surrounding “designer mixes,” health measures, and breed fads, were characterized as a structural rather than solely individual issue. These discrepancies between public perception and welfare science have been identified in previous studies of breeding ethics and purchasing behaviour [23].

Regulations and what it means to be “regulated” were frequently discussed by breeders as both a potential safeguard and a source of tension. Support for formal oversight of breeding was mixed. Qualitative responses illustrate that there may be mixed feelings towards regulations. Many breeders expressed concern that overregulation disproportionately impacts small-scale, ethical breeders. They described situations where complex or restrictive regulations make it harder for responsible hobby breeders to operate, while large-scale commercial operations continue to meet market demand regardless of legislative constraints. Several participants reasoned that ineffective enforcement and blanket legislation do little to prevent welfare risks from high-volume breeders or puppy mills who may operate illegally, meaning that the overall market pressures on dog welfare remain largely unchanged. These breeders often note that regulations, while intended to protect animal welfare, can disproportionately impact “responsible” breeders.

Although many breeders identified overregulation as a key challenge, the Canadian dog breeding sector is characterized by relatively limited formal regulation. Outside of municipal kennel licensing requirements, which many respondents do not appear to require due to home-based housing arrangements, there are few binding regulatory frameworks governing dog breeding in Canada. It is also possible that the prevalence of home-based breeding reflects, in part, the greater operational and regulatory burden associated with kennel-based systems. In this context, concerns about overregulation may represent a perceived or anticipatory threat rather than a response to existing regulatory constraints.

When participants were asked about their perspectives on breeder standards, they revealed further complexity. Notably, many respondents felt that current standards were poorly aligned with small-scale or home-based breeding environments. Standards developed primarily for kennel settings were often described as technically correct but contextually unrealistic. This mirrors observations in farm animal welfare research, where “one-size-fits-all” regulations may fail to capture the lived realities of heterogeneous management systems and having numerous standards can overload operators, making them difficult to understand or implement [24]. Participants feared that large-scale, biosecurity-focused standards may inadvertently compromise behavioural welfare in home settings, suggesting a need for more flexible, evidence-driven guidelines that account for environmental diversity.

When discussing regulating breeding, it is also important to remember that the breeders who participated in this study represent those willing to engage with research, breeders who, based on their reported practices, appear to already be operating at a high standard of care. As such, any development of regulation or legislation must be reflective of their experiences and values, ensuring that policy efforts do not inadvertently undermine those already striving to meet the welfare expectations and provide the humane care the public increasingly demands. At the same time, these findings should not be assumed to represent the full spectrum of breeding practices, particularly those operating below existing minimum standards. Regulatory approaches aimed at addressing substandard or harmful practices may therefore have impacts not captured in this sample and require evidence drawn from breeders who were not represented in this study.

Breeders frequently identified rising veterinary and care costs as barriers to maintaining welfare standards, highlighting the role of broader economic constraints in breeding practices. Research has repeatedly shown that access to veterinary services is uneven across communities, shaped by barriers such as financial limitations, distance to providers, limited service availability, and other structural constraints [25]. Because animal caretakers rely heavily on veterinarians for both medical care and welfare guidance, restricted access to veterinary services can have direct consequences for animal health and wellbeing. Studies have linked limited veterinary access to increased disease risk, poorer management practices, and overall compromised welfare outcomes [25], stressing why veterinary care remains a major concern across all animal-keeping contexts, including breeding. In fact, limited access to veterinary care represents a substantial and growing animal welfare concern in Canada, as unmet veterinary needs can compromise animal health, exacerbate preventable suffering, and strain the human–animal bond [26]. As such, access to care is increasingly recognized as a critical component of safeguarding companion animal welfare at a population level.

Ultimately, improving breeding dog welfare will require solutions that go beyond basic registration or fixed housing standards. Participants’ reflections indicate that welfare risks arise from the intersection of regulation, economics, knowledge, access to veterinary services, breed-specific health considerations, and buyer expectations. Responses revealed that breeders view dogs’ welfare not just as the result of individual husbandry decisions or breeder attitude but rather situated within broader social, economic, and regulatory structures. This identified complexity suggests that solutions aimed at improving welfare will need to be multifaceted and informed by breeder perspectives to support both adoption and impact. Future research should build on these findings by working collaboratively with breeders to address the systemic challenges they identified, including regulatory mismatch, economic pressures, and gaps in communication and knowledge. Incorporating the perspectives of other key stakeholders, particularly veterinarians and dog owners, would provide a more comprehensive understanding of breeder–buyer interactions, professional constraints, and the longer-term medical and behavioural outcomes associated with different breeding practices in Canada.

Several limitations should be considered when interpreting these findings. Recruitment relied heavily on kennel clubs, breed-specific groups, and voluntary participation, increasing the likelihood of self-selection bias and potentially overrepresenting breeders who are more engaged, welfare-oriented, or confident in their practices. Breeders operating outside formal networks, including large-scale commercial breeders or those breeding designer crossbreeds, were likely underrepresented. Additionally, all data were self-reported and may have been influenced by recall error or social desirability bias, particularly given the sensitive nature of animal welfare research and breeding ethics. Because the survey was distributed exclusively through online channels, breeders with limited internet access, those with lower levels of digital engagement, or those who do not participate in online breeder communities may have also been underrepresented in the sample. Importantly, this study assessed reported housing and management practices rather than directly measuring animal welfare outcomes, and therefore, conclusions regarding actual welfare status should be interpreted cautiously.

## 5. Conclusions

In conclusion, this study demonstrates that small-scale dog breeding in Canada is neither uniform nor easily defined by a single set of practices or values. Instead, it encompasses diverse approaches, as illustrated by the distinction between Independent Breeders and Structured Competition Breeders, alongside substantial shared commitments to providing social, enriched, and home-based care. While these breeders differ in their alignment with formal institutions, perceptions of risk, and definitions of responsibility, they converge on many core elements of welfare, particularly the importance of human interaction, behavioural enrichment, and maintaining dogs within domestic environments. These findings challenge simplified narratives that position breeders along a single ethical continuum and instead highlight the need to recognize variation within small-scale systems as a meaningful and influential component of the broader breeding landscape. At the same time, improving breeding dog welfare will require approaches that extend beyond existing frameworks centred on kennel club affiliation or standardized regulations. Breeders’ perspectives in this study demonstrate the importance of context-sensitive policies that balance welfare goals with feasibility, address structural challenges such as access to veterinary care and economic pressures, and incorporate the roles of both breeders and buyers in shaping outcomes. Importantly, the present findings are limited to self-reported practices, attitudes, and perceptions among the surveyed breeders and do not permit direct assessment of welfare outcomes. Furthermore, given the study’s recruitment methods and likely underrepresentation of some breeding populations, the results should be interpreted as reflecting the characteristics of the participating breeders rather than the Canadian dog breeding sector as a whole. Future research should directly assess welfare metrics in these environments, aim to capture underrepresented portions of the breeding community, particularly those operating outside formal networks, and further investigate how different regulatory, economic, and social conditions influence welfare practices. By integrating empirical evidence with stakeholder perspectives, more effective, inclusive, and practical strategies can be developed to support the welfare of breeding dogs across diverse systems.

## Figures and Tables

**Table 1:** Number (count) and percentage of respondents reporting housing type of breeding dogs not preparing for birth, birthing, or nursing.

Housing Type	Count	Percent (%)
Kennel housing	7	2.3
Home housing	252	83.4
Guardian or foster families	18	6.0
Other	25	8.3

**Table 2:** Comparison of data from the current study and existing data from commercial breeders on proportion of breeders who provide various forms of training, exercise, and enrichment to their breeding stock. For questions that were only used in this survey, the commercial operator value is left blank. Frequency responses in the current study were aggregated yes/no responses.

Category	Provided Enrichment	Percent (%) from Canadian Breeders: Current Study	Percent (%) from Commercial Operators: Stella et al. (2019)
Training	Potty/house training	96.5	
	Basic commands	95.2	0.0
	Tricks	57.1	0.0
	Sports	55.1	0.0
	Crate	83.0	
Exercise	Off-leash walks	70.9	
	Leashed walks	66.2	12.5
Enrichment	Play with humans	98.6	62.5
	Play with dogs	92.3	100.0
	Toys	95.9	56.3
	Chews	75.7	87.5
	Puzzle feeders	33.6	18.8
	Play with other animals	22.2	0.0

**Table 3:** Reported primary breeding purpose.

Breeding Purpose	Count	%
Companionship (e.g., pet)	266	92.0
Competition—conformation	224	77.5
Competition—performance (e.g., racing, agility, obedience, scent work, hunting, field trials)	222	76.8
Working—assistance (e.g., service, guide, assistance)	94	32.5
Working—on farm (e.g., guarding, herding)	44	15.2
Working—protection (e.g., military, detection, police)	25	8.7
Other	34	11.8

**Table 4:** Summary statistics for breeder rankings of dog welfare aspects.

Welfare Aspect	Mean	Median	SD
Physical Health	9.84	10	0.88
Mental Wellbeing	9.83	10	0.74
Temperament	9.74	10	0.77
Genetic Health	9.63	10	1.12
Conformation	8.70	9	1.66

**Table 5:** Summary statistics for breeder rankings of housing and welfare aspects.

Item	Mean	Median	SD
Cleanliness	2.48	2	1.39
Size/Space	3.15	3	1.27
Noise Control	5.53	6	0.89
Enriching Environment	3.08	3	1.21
Interaction with Humans	2.23	2	1.36
Interaction with Dogs	4.53	5	1.32

**Table 6:** Fit statistics for unconditional LCA for models with 1 to 5 classes (AIC = Akaike information criterion; BIC = Bayesian information criterion).

Number of Classes	Max Log-Likelihood	AIC	BIC	Adjusted BIC	Corrected AIC	Entropy
1	−1687.14	3400.29	3450.26	3409.02	3401.39	∞
2	−1690.84	3435.69	3539.47	3453.81	3440.46	0.669
3	−1684.29	3450.59	3608.17	3478.11	3461.95	0.615
4	−1672.94	3455.88	3667.28	3492.80	3477.20	0.518
5	−1670.62	3479.25	3744.45	3525.57	3514.38	0.550

## Data Availability

The original data presented in the study are openly available in Borealis at https://doi.org/10.5683/SP3/85H27N.

## Abbreviations

The following abbreviations are used in this manuscript:

BC	British Columbia, Canada
CAPTCHA	Completely Automated Public Turing test to tell Computers and Humans Apart
CVMA	Canadian Veterinary Medical Association
CKC	Canadian Kennel Club
FCI	Fédération Cynologique Internationale
FIML	Full-Information Maximum Likelihood
HCC	Hair Cortisol Concentration
IQR	Interquartile Range
LCA	Latent Class Analysis
ON	Ontario, Canada
SLO	Social Licence to Operate
US	The United States of America

## Appendix A. Survey Tool

Q1 Please check the box below (CAPTCHA)
Q3 Which of the following best describes your involvement in dog breeding over the past three years?

- My dog got pregnant and had a litter of puppies but it was unplanned
- I have one or more breeding dams that I purposely breed
- I breed or have allowed only my male (sire) to breed (excluding dams)
- I have not bred dogs

*Size and Management*
Q4 How many adult active breeding females (females that you have bred and/or plan on breeding, not including retired females) have you owned per year in the past 3 years? *Include all the dogs you’ve owned, even those you bred that did not live with you*. (Please answer in numbers)

- 2024 __________________________________________________
- 2023 __________________________________________________
- 2022 __________________________________________________

Q5 How many adult intact males have you owned per year in the past 3 years? *Include all the dogs you’ve owned, even those you bred that didn’t live with you*. (Please answer in numbers)

- 2024 __________________________________________________
- 2023 __________________________________________________
- 2022 __________________________________________________

Q6 How many retired dogs have you owned per year in the past 3 years? *Include all the dogs you’ve owned, even those you bred that didn’t live with you.* (Please answer in numbers)

- 2024 __________________________________________________
- 2023 __________________________________________________
- 2022 __________________________________________________

Q7 How many litters have you had per year in the past 3 years? (Please answer in numbers)

- 2024 __________________________________________________
- 2023 __________________________________________________
- 2022 __________________________________________________

Q8 What type of records do you keep?

○ Tracking food intake and/or elimination
○ Weight trends
○ Dewormer & Flea/tick treatments
○ Genetic records
○ Vaccine records
○ Reproductive history
○ Observation sheets for behaviour
○ Import & Export records
○ Sales records
○ Other __________________________________________________

Q9 How many different dog breeds or crossbreeds have you bred at your facility in the past three years? (Please answer in numbers)
________________________________________________________________

Q10 Is your primary breed a crossbreed?

- No
- Yes

Q11 Please select the breed or cross-breed that you have been most involved with breeding over the past three years. Please answer the rest of the survey about only this breed.

▼ Mixed Breed (1) … Other (217)

Q12 If your primary breed was not listed, please write in or describe the breed below. Please answer the rest of the survey about only this breed. If your primary breed was listed, please leave this question blank.
________________________________________________________________

Q13 Are you a member of any of the following breed clubs?

○ Canadian Kennel Club
○ Breed Specific groups (e.g., GANA)
○ Local breeding networks
○ Other __________________________________________________
○ No

Q14 Does each dam or sire undergo genetic testing?

- Yes
- Only if there is an issue with the dam’s or sire’s offspring
- No

Q15 Which of the following breeding techniques do you use? Please select all that apply.

○ Naturally, with supervision
○ Naturally, without supervision
○ Transvaginal insemination
○ Transcervical insemination
○ Surgical insemination
○ Other (please specify) __________________________________________________

Q16 For which of the following reasons are your female dogs seen by a veterinarian? Please select all that apply.

○ Emergency examinations when sick or injured
○ Examinations and monitoring when pregnant and healthy
○ Examinations and monitoring when pregnant with health issues
○ Routine preventive wellness examinations (outside of pregnancy)
○ Legally required vaccinations
○ Optional vaccinations
○ Other __________________________________________________

Q17 Do you follow pre-established standards of care or generally accepted management practices for housing and raising dogs? Please select all that apply

○ CKC By-Laws
○ CVMA Code of Practice for Canadian Kennel Operatations
○ I utilize my own experience in designing my housing and care program
○ Other (please specify) __________________________________________________

Q18 In your opinion, are there limitations to current standards that are difficult or illogical to follow? If yes, please describe how.
______________________________________________________________

Q19 How strongly do you agree with these limitations to increasing the size of your operation?

	Strongly disagree	Somewhat disagree	Neither agree nor disagree	Somewhat agree	Strongly agree
Desire (only want to breed the number of dogs you have)					
Physical space/facility (number of dogs that can be reasonably housed in your current set up)					
Time (don’t have enough time to care for or arrange the breeding of more dogs)					
Resources (too expensive or would cost more than it is worth to breed more dogs)					
Regulations (city bylaw or provincial regulations that limit the number of dogs in your set-up)					
Other (please explain)					
Desire (only want to breed the number of dogs you have)					

Q20 What percentage of buyers view your facility and dogs in person before purchasing puppies?

	0	10	20	30	40	50	60	70	80	90	100
Percent (%) ()											
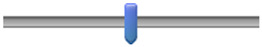

Q21 What challenges arise with having the buyers come to your facility or see your adult dogs?
_____________________________________________________________

Q22 Are there any circumstances in which you would not accept the return of a dog? If yes, please list possible circumstances

- Yes __________________________________________________
- No

*Housing and Daily Care*
Q23 How many caregivers (family, friends, staff etc.) care for your dogs on a regular basis? Please include yourself in the total.

▼ 1 (1) … 10 (10)

Q24 How would you categorize how you manage your breeding dogs who are not currently preparing for birth, birthing or nursing?

- Kennel housing
- Home housing
- Guardian or foster families
- Other __________________________________________________

Q25 What best describes the environment in which your breeding dogs spend most of their time?

- An area with free access to humans (e.g., in the home as pets)
- An area with frequent access to humans (e.g., free ranging in the yard or garage/barn)
- An area where they have controlled access to humans (e.g., a kennel, run, or crate)

Q26 Where do your dams whelp (give birth)?

- In my house
- In a building that I do not live in
- Outside on my property with shelter
- At another location under the management of others (e.g., foster home)
- At a veterinary clinic
- Other __________________________________________________

Q27 Where do you house your dams and litters during nursing?

- In my house
- In a building that I do not live in
- Outside on my property with shelter
- At another location under the management of others (e.g., foster home)
- Other (please specify) __________________________________________________

Q28 Once the puppies do not need constant supervision, can the dam voluntarily leave the puppies when she is with them (e.g., through a gate or out of the box)?

- Yes, freely at all times
- Yes, at certain times
- No

Q29 Do your breeding females leave the property on a regular basis for enrichment (e.g., on walks, at the park etc.)? If no, please list some reasons you might choose not to.

- Yes
- No __________________________________________________

Q30 What types of training do you include in your dogs’ routine? Please select those that apply.

	Daily	2–3 times a week	Once a week	Once a month or less	Never
Leashed walks					
Off-leashed walks					
Basic commands (e.g., come or sit)					
Tricks (e.g., shake)					
Sport or professional related training (e.g., agility, conformation, or therapy dog related commands)					
Crate training					
Potty/House training					
Other					

Q31 What enrichment features do you include in your dogs’ routine or housing? Please select those that apply.

	Daily	2–3 times a week	Once a week	Once a month or less	Never
Puzzle feeder (e.g., kong with wet food)					
Chews (e.g., bone)					
Toys					
Free roaming outdoor time (not including potty breaks)					
Play with other dogs					
Play with humans					
Play with other animals (non-dogs)					
Other (please explain)					

Q32 In months, what is a typical age range that you begin breeding your dams?
_______ Minimum number of months
_______ Maximum number of months of age range

Q33 How frequently do your dams require medical intervention during birth (e.g., induction, cesarean sections etc.)?

- Never
- Sometimes
- About half the time
- Most of the time
- Always

Q34 On average, how soon after having a litter do you breed a dam again?

▼ First heat after whelping (1) … Other (11)

Q35 For what reason do you retire dams from breeding? Please select all that apply.

○ After having a certain number of litters. (Please specify how many)
  __________________________________________________
○ Behavioural, medical or genetic issues
○ On advice from a veterinarian
○ At a certain age
○ I do not retire dams
○ Other (please specify) __________________________________________________

Q36 Do you sell or rehome your retired animals?

- Yes
- No
- Other __________________________________________________

*Attitudes and Values*
Q37 What is the intended purpose of the dogs that you breed? Please select all that apply.

○ Companionship (e.g., pet)
○ Working—assistance (e.g., service, guide, assistance)
○ Working—on farm (e.g., on farm guarding and herding)
○ Working—protection (e.g., military, detection, police)
○ Competition—Conformation
○ Competition—Performance (e.g., racing, agility, obedience, scent work, hunting, field trials)
○ Other __________________________________________________

Q38 Please rank how important you feel these aspects are for the welfare of the dogs in your care

	0	1	2	3	4	5	6	7	8	9	10
Dogs’ basic physical health ()											
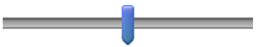
Dogs’ mental wellbeing ()											
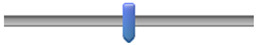
Genetic health ()											
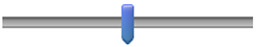
Conformation (e.g., coat type, size) ()											
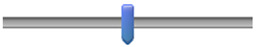
Temperament (e.g., sociability, drive, intelligence) ()											
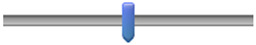

Q39 Can you list some practical ways you provide good welfare for your dogs?
_____________________________________________________________

Q40 Please rank how important you feel these aspects are for the welfare of the dogs in your care by dragging and dropping the labels.
______ Facility cleanliness
______ Size/space for dogs
______ Noise control
______ Enriching environment
______ Ability to interact with humans
______ Ability to interact with other dogs

Q41 How strongly do you agree with the following statements

	Strongly disagree	Disagree	Somewhat disagree	Neither agree nor disagree	Somewhat agree	Agree
Animals (including dogs) possess moral status, i.e., that they have interests that need to be taken into account						
Animal based operations should be monitored by an independent 3rd party to make sure animals are being properly cared for						
I am supportive of regulations for breeding						
Legislation would improve the conditions for bred dogs						
Legislation would improve the conditions for breeders						
Legislation would improve outcomes for dog buyers						

Q42 Previous research has shown that pet owners have varying perspectives on the intelligence of their pets. These questions have not previously been asked of pet breeders. Please use the slider below to indicate how intelligent and sentient (aware or conscious) you think dogs are, on a scale from 0 (like an inanimate object) to 10 (like a human). If you do not feel comfortable answering, please select “prefer not to answer”.

Inanimate object (0)				Human (10)				Prefer not to answer		
0	1	2	3	4	5	6	7	8	9	10
Dogs ()										
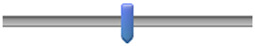

Q43 What do you think is the biggest risk to dog welfare in the dog breeding industry?
________________________________________________________________

Q44 What measures do you take to avoid these issues and risks to your dogs?
________________________________________________________________

*Demographics*
Q45 Where are you located? Please answer as much as you feel comfortable sharing.

○ Country __________________________________________________
○ Province __________________________________________________
○ City __________________________________________________
○ Prefer not to answer

Q46 I live in a:

- Rural property outside of a concentrated housing area
- Village (<1000 people)
- Small town (1000 to 20,000 people)
- Large town (20,000 to 100,000 people)
- Small city (100,000 to 300,000 people)
- Large city (300,000 to 1 million people)
- Metropolis (>1 million people)
- Prefer not to answer

Q47 What best exemplifies the type of dwelling where you reside?

- Apartment
- Townhouse
- Detached House
- Acreage
- Mobile home/Trailer
- Other __________________________________________________
- Prefer not to answer

Q48 Do you have a profession outside of dog breeding?

- Yes
- No

Q49 Is your profession in the animal industry?

- Yes
- Other __________________________________________________
- No

Q50 What is your annual household income?

- Below $20,000
- $20,000–$40,000
- $40,000–$60,000
- $60,000–$80,000
- $80,000–$100,000
- Above $100,000
- Prefer not to answer

Q51 Do you receive any government assistance?

- Yes
- No
- Prefer not to answer

Q52 What is your age? Please select from the drop down below.

▼ 1 (1) … 105 (105)

Q53 What is your highest level of education

- 8th grade
- Highschool diploma or GED
- Trade/technical/vocational training
- Associate degree
- Bachelor’s degree
- Masters degree
- Professional degree
- Doctorate degree
- Other __________________________________________________
- Prefer not to answer

Q54 Do you have any formal training or education in animal husbandry, medicine or breeding?

- Yes
- No

Q55 Do you have any formal training or education in animal training?

- Yes
- No

Q56 How many years’ experience do you have breeding dogs?

▼ 1 (1) … 100 (100)

Q57 Do you identify as Indigenous?

- Yes
- No
- Prefer not to answer

Q58 Do you identify as a member of a racialized group? (Persons other than indigenous peoples, who are non-caucasian in race or non-white in colour. Consisting mainly of the following groups: South Asian, Chinese, Black, Filipino, Latin American, Arab, Southeast Asian, West Asian, Korean and Japanese).

- Yes
- No
- Prefer not to answer

*Email*
Q59 If you would like to be contacted to participate in follow up dog breeding studies, please follow the link below. If you do not want to participate in future research please press the forward arrow.

## Appendix B. Qualitative Codebooks

### Appendix B.1. Codebook 1

**Table A1:** Codebook.

Code	Subcode	Description	Example Quote	Counts
**Animal advocacy**		Quotes related to individual or organizational advocacy for animal rights.	“Animal rights activists that take things to extremes”	170
	*Adopt don’t shop*	Quotes related to animal advocacy surrounding the ‘adopt don’t shop’ movement.	“rescue’s—adopt rather than shop mentality—those that support adoption over purchasing from a reputable ethical breeder”	5
	*Dogs should not be pets*	Quotes related to animal advocacy for dogs not being kept as pets.	“do gooders who think dogs should not be pets, PETA is horrible danger to dogs.”	3
**Ineffective regulations or legislation**		Quotes related to breeder regulations being ineffective to ensure good dog welfare.	“Sweeping regulations being made that don’t consider the ethical breeder perspective and instead focus solely on puppy mills and backyard breeders”	60
	*Insufficient regulation or enforcement*	Quotes related to regulations not effectively stopping harmful breeding practices.	“It is completely unregulated, but I do not believe regulating would change anything because it would only work on a complaint system.” “Regulations that… don’t prevent bad breeders and bad practices.” “regulations have been… easier for large scale breeders (puppy mills & BYB) to operate and be seen as “approved” or “certified” because they meet livestock type breeding regulations.”	9
	*Overregulating or legislating ‘good’ breeders*	Quotes related to regulations unnecessarily hindering non-harmful breeding practices.	“Over imposing legislation that is restrictive to home based preservation breeders” “Regulations have been seen to make it harder for ethical, hobby breeders to operate…”	11
**Extreme conformation in breed standards**		Quotes related to extreme conformation that are associated with welfare issues in kennel club breed standards.	“Dog shows creating standards that are unhealthy for the dog, like German Shepherds having back issues or Brachycephalic dogs who cant breathe well or give birth naturally.”	4
**Conflict between breeders**		Quotes related to conflict between breeders surrounding to breeding practices, experience or beliefs.	“dog shows creating standards that are unhealthy… yet breeders hate doodle breeders who are breeding healthy dogs. So much hate.”	2
**Breeders prioritizing profit**		Quotes related to commercialization of dog breeding, prioritizing making a profit or supplying to meet public demand over breed preservation or dog welfare.	“breeders that are breeding for profit and strictly as a business vs. wanting to preserve, maintain and better the breed.”	170
	*Greed*	Quotes related to breeders being motivated by greed.	“Greed. People who breed for money and do not care about the dogs.”	6
	*High volume operations*	Quotes related to breeders producing large numbers of puppies by having large breeding operations.	“breeders who breed high volumes for profit with little regard for dogs” “people get an operation that becomes to big to handle. They don’t have the staff or the room to consider the dogs welfare.”	6
	*Overbreeding*	Quotes related to breeders overbreeding individual females or breeding beyond their capacity or buyer pool.	“overbreeding” “overbreeding beyond the need for dogs”	14
	*‘Backyard breeders’ or ‘puppy mills’*	Quotes related to ‘backyard breeders’ or ‘puppy mills’	“puppy mills, breeding solely for profit” “puppy mills are a huge issue”	50
**Overpopulation**		Quotes related to there being too many dogs for the number of caretakers.	“over population” “overbreeding beyond the need for dogs”	4
**Poor breeding stock selection**		Quotes related to dog breeders not adequately selecting appropriate breeding stock.	“Breeding dogs that are not healthy and do not have sound, stable temperament.”	156
	*Crossbreeds and ‘designer mixes’*	Quotes related to dog breeders cross breeding to create new unregistered breeds.	“Cross breeding of dogs”	36
	*Failure to test or incomplete genetic and/or health testing*	Quotes related to breeders not completing adequate genetic or health testing for breeding stock selection.	“Breeding non health tested dogs”	43
	*Ignoring known hereditary concerns*	Quotes related to breeders knowingly continuing to use breeding stock with hereditary concerns.	“knowingly breeding a genetic health issue”	2
**Lack of accurate knowledge or understanding**		Quotes related missing or incorrect information or understanding.	“Uneducated people” “inaccurate information being shared” “lack of education (on many fronts)”	131
	*False marketing from breeders/rescues*	Quotes related to breeders or rescues misleading buyers about the origin or state of puppies being sold.	“Fake rescues who take advantage of buying poorly bred dogs to sell” “Good advertising of poor breeders who are outnumbering good breeders…”	16
	*Uninformed breeders*	Quotes related to breeders who lack breed-specific knowledge or don’t know enough about breeding to do it ‘responsibly’.	“Uneducated… breeders” “breeders who are not prepared”	13
	*Uninformed buyers*	Quotes related to buyers not knowing how to identify ‘good breeding practices’ or how to care for dogs.	“Lack of knowledge of people buying dogs.” “uneducated… buyers” “buyers generally falling for fad words such as “genetically tested”, “rare”, etc. when pertaining to a purchase” “lack of informed buyers” “buyers no longer understand what the responsibilities of having a dog are.”	23
	*Public attitudes*	Quotes related to inaccurate or misinformed public attitudes that shape supply and demand of dogs.	“The continuing myth that “its all how you raise them” which allows buyers to feel justified in acquiring puppies from less than ethical resources without adequate research into breed/lines traits” “breed fads”	11
	*The term industry*	Quotes related to issues with the term industry to describe dog breeding or explaining that it is not an industry.	“The word industry. It’s a vocation in my opinion”	4
**Importing dogs**		Quotes related to dogs being imported from other countries to be sold in Canada.	“unethical and dangerous importation of “rescue” dogs” “importation from third world countries with disease and faked documentation.” “importing of dogs from countries that have had hurricanes etc.”	9
**Lack of access to veterinary care**		Quotes related to the lack of access to veterinary care for breeders.	“lack of essential vet care”	33
	*Reproductive specialists*	Quotes related to the lack of access to veterinary specialists in dog reproduction.	“veterinarian not schooled enough for breeding practices and fertility. We need more repro vets out there!”	3
	*Cost*	Quotes related to the cost of veterinary care being too high.	“vet care and cost of services is becoming a huge barrier for a lot of breeders…”	11
**Failure to provide adequate daily care**		Quotes related to dog caretakers not providing adequate daily care to dogs.	“lack of care”	52
	*Lack of appropriate nutrition*	Quotes related to dog caretakers not providing appropriate nutrition.	“low quality food” “feeding processed kibbles such as Purina, Hills science etc.”	9
	*Lack of stimulation or enrichment*	Quotes related to dog caretakers not providing appropriate stimulation or enrichment.	“Stimulation” “lack of human interaction, lack of social enrichment, lack of training, rules and boundaries.” “lack of interaction with humans, lack of socialization, lack of variety and enrichment in their daily lives”	13
	*Lack of appropriate housing or environment*	Quotes related to dog caretakers not providing the appropriate environment and housing.	“poor cleanliness” “poor living conditions”	10
**Breeding dog welfare**		Quotes related to breeding dog welfare being compromised.	“lack of quality of life in the breeding dogs whether it be physical or mental” “issues during delivery” “happy non-stressed dam have more resilient pups and I don’t think a dam can not be stressed out in a facility”	10
**Failure to commit to a dog for their whole life**		Quotes related to dog caretakers not committing to support a dog for the duration of their life.	“people who discard dogs” “dog ending up abused, untrained and unwanted”	32
	*Breeders not taking a dog back*	Quotes related to breeders who do not take dogs back when buyers can no longer keep them.	“breeders who don’t accept a dog back for any reason” “backyard breeders dumping their puppies” “no lifetime breeder support, refusing to take back a pup/dog for any reason”	9
	*Dogs ending up in shelters*	Quotes relating to bred dogs ending up in shelters.	“…purpose bred litters… sold to buyers who will give their all to their puppy and with no chance of pups ending up in a shelter or abandon” “Breeding dogs that are not healthy… these dogs end up in shelters as the owners are not able to properly care for them…”	8
**Economy**		Quotes related to the overall economic pressure on caretakers	“A failing economy (people cannot afford to feed themselves often cut back on “luxuries” such as vet care and nutritious pet food, etc.)	2
**Complexity**		Quotes related to the complexity of factors impacting dog welfare.	1	
**Uncoded**		Quotes that are unclear of difficult to categorize without making assumptions.	23	

### Appendix B.2. Codebook 2

**Table A2:** Codebook.

Code	Subcode	Description	Example Quote	Counts
**Standards made for kennel operations are not applicable for home breeders**		Quotes related to standards being designed to regulate large or commercial and kennel operations and are not applicable for small breeders whose dogs live in the home.	“Many are based on commercial breeding farm set ups vs. single litters” “Standards are designed for large scale operations. Not all are necessarily practical or necessary when the animals live in the home”	34
	*Prioritizing biosecurity over comfort or behaviour*	Quotes related to standards prioritizing biosecurity in large facilities that isn’t as relevant in small, home breeders.	Some with regard to hard surfaces for cleanliness. Dogs in my home are on dog beds and blankets—washed as needed” “my dogs are in my house, surfaces are of course penetrable” “Standards describe puppies growing up in conditions which can be entirely sanitized dog not account for the puppy’s need to be able to do species specific behaviours… can only lead to sub-standard behavioural health”	3
	*Some standards are challenging to follow*	Quotes related to standards being difficult to follow for home breeders.	“CVO makes many husbandry practices difficult”	3
**One-size-fits-all standards are not always applicable**		Quotes related to standards being one-size-fits all which leads to them not being applicable in all scenarios.		19
	*Blanket breed or practice bans*	Quotes related to blanket breed or practice bans.	“The provincial ban on cropping and docking is very very harmful to the state of purebred dogs in Canada” “I am more concerned about banning my breed for so-called breathing issues because there’s too many backyard breeders that have to answer to no one”	5
	*Breed, litter and individual differences not accounted for*	Quotes related to standards not accounting or allowing for individual, litter and breed differences.	“There are differences in each breed and each realm of breeding that are not accounted for.”	4
**Municipal bylaw constraints**		Quotes related to municipal bylaw constraints.		42
	*Dog number limits*	Quotes related to municipal limits to dogs on one property.	“Municipal law does not allow having more than 3 adult dogs in the home even with a kennel licence”	14
	*Kennel licencing*	Quotes related to municipal constraints on kennel licencing.	“I wish it was easier to obtain a kennel license when living in town” “kennel licence requires dogs not be housed in the home.”	5
**Import/export restrictions**		Quotes related to standards including restrictions on importing and exporting of dogs.	“CDC recent limitations to importing puppies into the USA”	6
**Requirements without accessibility**		Quotes related to standards having requirements that are not easily complied with due to existing barriers.		5
	*Cost*	Quotes related to cost being a barrier to comply with regulations.	“Expenses have gone through the roof, vet costs/care puppy vaccines care of the dam and all the dogs etc.”	1
	*Availability*	Quotes related to availability of necessary expertise, appointments or equipment being a barrier to comply with regulations.	“limited availability for appointments for recommended OFA testing”	2
**Standards don’t do enough**		Quotes related to the standards needing to be higher or enforceable in more situations.		11
	Do not restrict “backyard breeders”	Quotes related to standards not being applied to “backyard breeders”	“I hate how we as CKC members are bound by bylaws and observed rules that are governed by the agriculture minister however those rules never apply to backyard breeders”	3
	Not high enough	Quotes related to standards lacking depth or not being detailed or high enough.	“They should be more in depth, more care is needed” “I find the standards aren’t high enough. The CKC still allows dogs to be housed in crates and kennels and no inspections are completed by the organization. Standards need to be raised!”	2
**Standards are incorrect**		Quotes related to standards recommending incorrect management practices.	“breeding bitches should be bred back to back then skip”	1
**Criticism of organizations who create standards**		Quotes related to criticism of organizations like CVMA and CKC who create standards.	“CKC are a bunch of ridiculous hypocrites with their heads in the sand”	7
**Lack of understanding**		Quotes related to a lack of understanding of the participant.		18
	*Have not read existing standards*	Quotes related to not having read or been aware of existing standards.	“I honestly havent even looked at the CVMA standards”	4
	*Unclear which standards the question refers to*	Quotes related to a lack of understanding of what standards the question was referring to.	“Do you mean breed standards?” “I don’t know the “standards” you refer to”	4
**No limitations of standards**		Quotes related to there being no limitations to existing breeding standards.	“no, I don’t think there are limitations”	50
**Uncoded**		Quotes that are unclear of difficult to categorize without making assumptions.	“colour that is naturally occurring in the breed that is has no health issues attached and nothing more than visual”	6

## Appendix C. Complete Breed List

**Table A3:** All breeds selected from question 11 “Please select the breed or cross-breed that you have been most involved with breeding over the past three years. Please answer the rest of the survey about only this breed”.

Breed	n	Breed	n	Breed	n
Retriever (Labrador)	27	Australian Shepherd	13	Retriever (Golden)	11
Retriever (Nova Scotia Duck Tolling)	9	Belgian Shepherd Dog	8	Border Terrier	8
German Shepherd Dog	8	Samoyed	7	Doberman Pinscher	6
Great Dane	6	Keeshond	6	Poodle (Standard)	6
Rottweiler	6	Welsh Corgi (Pembroke)	6	Whippet	6
Bernese Mountain Dog	5	Poodle (Miniature)	5	Weimaraner	5
Bulldog	4	Dachshund (Miniature Long-Haired)	4	Mixed Breed	4
Newfoundland	4	Papillon	4	Retriever (Flat-Coated)	4
Shiba Inu	4	Vizsla (Smooth-Haired)	4	Barbet	3
Border Collie	3	Bull Terrier (Miniature)	3	Bullmastiff	3
Cavalier King Charles Spaniel	3	Fox Terrier (Smooth)	3	Irish Wolfhound	3
Labradoodle	3	Mastiff	3	Old English Sheepdog	3
Pomeranian	3	Schnauzer (Miniature)	3	Shetland Sheepdog	3
Siberian Husky	3	Alaskan Malamute	2	American Staffordshire Terrier	2
Australian Cattle Dog	2	Beagle	2	Bearded Collie	2
Bosten Terrier	2	Bouvier des Flandres	2	Boxer	2
Bull Terrier	2	Chihuahua (Short Coat)	2	Dalmation	2
Dandie Dinmont Terrier	2	Deerhound (Scottish)	2	Eurasier	2
French Bulldog	2	Goldendoodle	2	Greyhound	2
Lagotto Romagnolo	2	Leonberger	2	Manchester Terrier	2
Norwich Terrier	2	Pointer (German Short-Haired)	2	Pug	2
Spaniel (American Cocker)	2	Staffordshire Bull Terrier	2	Yorkshire Terrier	2
Airedale Terrier	1	Ausiedoodle	1	Bernedoodle	1
Bichon Firse	1	Bloodhound	1	Braque Francais Pyrenees	1
Cairn Terrier	1	Chinese Crested Dog	1	Chow Chow	1
Collie (Rough)	1	Coton de Tulear	1	Dachshund (Miniature Wire-Haired)	1
Finnish Lapphund	1	Finnish Spitz	1	Fox Terrier (Wire)	1
Griffon (Bruseels)	1	Havanese	1	Iceland Sheepdog	1
Italian Greyhound	1	Japanese Spitz	1	Miniature Pinscher	1
Mudi	1	Norrbottonspets	1	Norwegian Lundehund	1
Parson Russell Terrier	1	Pekingese	1	Petit Basset Griffon Vandeen	1
Pointer (German Wire-Haired)	1	Portuguese Water Dog	1	Retriever (Chesapeake Bay)	1
Rhodesian Ridgeback	1	Saluki	1	Schnauzer (Giant)	1
Setter (Gordon)	1	Setter (Irish Red and White)	1	Shapendoes	1
Sheepadoodle	1	Shih Tzu	1	Silky Terrier	1
Soft-Coated Wheaten Terrier	1	Spaniel (English Cocker)	1	Spaniel (Irish Water)	1
Spaniel (Welsh Springer)	1	Spanish Water Dog	1	Tibetan Terrier	1
West Highland White Terrier	1			Other	12
				Missing/blank	11

## Appendix D. Additional Results

**Table A4:** Number (count) and percentage of dog breeders surveyed by province.

Province	Count	Percent (%)
British Columbia	81	26.5
Ontario	64	20.9
Alberta	46	15
Saskatchewan	9	2.9
Manitoba	6	2
Nova Scotia	6	2
Quebec	6	2
New Brunswick	5	1.6
Prince Edward Island	1	0.3

**Table A5:** Number (count) and percentage of dog breeders by type and size of community.

Community Size	Count	Percent (%)
Rural property outside of a concentrated housing area	148	57.1
Small town (1000 to 20,000 people)	33	12.7
Small city (100,000 to 300,000 people)	26	10
Large town (20,000 to 100,000 people)	18	7.1
Large city (300,000 to 1 million people)	12	4.6
Metropolis (>1 million people)	8	3.1
Village (<1000 people)	8	3.1
Prefer not to answer	4	1.5

**Table A6:** Number (count) and percentage of dog breeders by dwelling type.

Dwelling Type	Count	Percent (%)
Acreage	133	51.4
Detached house	106	40.9
Mobile home/trailer	2	0.8
Townhouse	2	0.8
Apartment	1	0.4
Prefer not to answer	1	0.4
Other	14	5.4

The majority reported having another profession (*n* = 214, 83.9%) outside of breeding, while a smaller proportion did not have work outside of breeding (*n* = 41, 16.1%).

Breeders who reported having a profession outside of breeding indicated that their profession was not in the animal industry (*n* = 152, 67.9%), while 72 breeders (32.1%) reported that their profession was related to the animal industry.

The largest proportion of breeders reported an income above $100,000 CAD (*n* = 98 breeders, 38.3%). Smaller proportions reported incomes between $80,000 and $100,000 (*n* = 34, 13.3%), $60,000 and $80,000 (*n* = 32, 12.5%), and $40,000 and $60,000 (*n* = 19, 7.4%). The remaining counts and percentages are listed in Table A7.

**Table A7:** Number (count) and percentage of dog breeders surveyed by annual household income.

Household Income (In CAD)	Count	Percent (%)
Above $100,000	98	38.3
$80,000–$100,000	34	13.3
$60,000–$80,000	32	12.5
$40,000–$60,000	19	7.4
$20,000–$40,000	15	5.9
Below $20,000	4	1.6
Prefer not to answer	54	21.1

The majority of breeders reported that they did not receive government assistance (*n* = 227, 88.7%). A small number indicated that they did receive assistance (*n* = 14, 5.5%), and 15 breeders (5.9%) preferred not to answer.

The most reported education levels were a Bachelor’s degree (*n* = 77, 30.1%) and trade, technical, or vocational training (*n* = 63, 24.6%). The remaining counts and percentages are listed in Table A8.

**Table A8:** Number (count) and percentage of dog breeders surveyed by highest education level.

Highest Education	Count	Percent (%)
Bachelor’s degree	77	30.1
Trade/technical/vocational training	63	24.6
Professional degree	28	10.9
High school diploma or GED	26	10.2
Master’s degree	17	6.6
Associate degree	16	6.2
Doctorate degree	8	3.1
Other	11	4.4
Prefer not to answer	10	3.9

The majority of breeders reported that they did not identify as Indigenous (*n* = 240, 94.1%). Six breeders (2.4%) reported that they did identify as Indigenous, and nine breeders (3.5%) preferred not to answer.

Most breeders also reported that they did not identify as part of a racialized group (*n* = 227, 90.8%). Seven breeders (2.8%) reported that they did, and 16 breeders (6.4%) preferred not to answer.

**Table A9:** Typical timing of breeding dams after whelping.

Timing of Next Breeding	Count	Percent (%)
First heat after whelping	29	10.0
Second heat after whelping	111	38.4
Third heat after whelping	32	11.1
Fourth heat after whelping	12	4.2
Fifth heat after whelping	2	0.7
Sixth heat after whelping	0	0.0
Seventh heat after whelping	1	0.3
Eighth heat after whelping	0	0.0
No particular time/plan freely	80	27.7
I don’t plan breeding	1	0.3
Other	21	7.3
